# RAGE displays sex-specific differences in obesity-induced adipose tissue insulin resistance

**DOI:** 10.1186/s13293-022-00476-6

**Published:** 2022-11-08

**Authors:** Zuoqin Du, Jiaqi Wu, Ziqian Feng, Xiaoyu Ma, Tao Zhang, Xin Shu, Jin Xu, Liqun Wang, Mao Luo, Jianbo Wu

**Affiliations:** 1 Drug Discovery Research Center, Laboratory for Cardiovascular Pharmacology, Department of Pharmacology, School of Pharmacy, Southwest Medical University, Luzhou, 646000 Sichuan People’s Republic of China; 2 Metabolic Vascular Disease Key Laboratory of Sichuan Province, Luzhou Municipal Key Laboratory of Thrombosis and Vascular Biology, Luzhou, 646000 Sichuan People’s Republic of China; 3 Key Laboratory of Medical Electrophysiology, Ministry of Education, Institute of Cardiovascular Research of Southwest Medical University, Luzhou, 646000 People’s Republic of China; 4 Zhengzhou Shuqing Medical College, Zhengzhou, 450064 People’s Republic of China

**Keywords:** Obesity, Receptor for advanced glycation end products (RAGE), Sex, Insulin resistance, Adipose tissue

## Abstract

**Background:**

The receptor for advanced glycation end products (RAGE) plays an important role in obesity-associated insulin sensitivity. We have also previously reported that RAGE deficiency improved insulin resistance in obesity-induced adipose tissue. The current study was aimed to elucidate the sex-specific mechanism of RAGE deficiency in adipose tissue metabolic regulation and systemic glucose homeostasis.

**Methods:**

RAGE-deficient (RAGE^−/−^) mice were fed a high-fat diet (HFD) and subjected to glucose and insulin tolerance tests. Subcutaneous adipose tissue (sAT) was collected, and macrophage polarization was assessed by quantitative real-time PCR. Immunoblotting was performed to evaluate the insulin signaling in adipose tissues.

**Results:**

Under HFD feeding conditions, body weight and adipocyte size of female RAGE deficient (RAGE^−/−^) were markedly lower than that of male mice. Female RAGE^−/−^ mice showed significantly improved glucose and insulin tolerance compared to male RAGE^−/−^ mice, accompanied with increased M2 macrophages polarization. Expressions of genes involved in anti-oxidant and browning were up-regulated in adipose tissues of female RAGE^−/−^ mice. Moreover, insulin-induced AKT phosphorylation was significantly elevated in adipose tissue in female RAGE^−/−^ mice compared to male RAGE^−/−^ mice.

**Conclusions:**

Our findings suggest that RAGE-mediated adipose tissue insulin resistance is sex-specific, which is associated with different expression of genes involved in anti-oxidant and browning and insulin-induced AKT phosphorylation.

**Supplementary Information:**

The online version contains supplementary material available at 10.1186/s13293-022-00476-6.

## Highlights

- Female RAGE^−/−^ mice showed significantly improved glucose and insulin tolerance compared to male RAGE^−/−^ mice.
- Female RAGE deficiency promotes M2 macrophage polarization in adipose tissues.
- Female RAGE deficiency prevents oxidative stress in adipose tissues.
- Female RAGE deficiency protects insulin-AKT signaling in adipose tissues.

**Supplementary Information:**

The online version contains supplementary material available at 10.1186/s13293-022-00476-6.

## Introduction

It is well established that estrogen regulates the metabolic status of white adipose tissue (WAT) in females, but the mechanisms underlying this phenotype remain unknown. There are dominant sex differences in the association between adipose tissue distribution, insulin sensitivity, and the development of type II diabetes [1, 2]. Although the relationship between adipose tissue and glucose homeostasis is well known, the role of sex difference and WAT in that relationship is far less defined.

Advanced glycation end products (AGEs) interact with their receptors (RAGE) in adipocytes [3]. RAGE was expressed in adipose tissues, which is down-regulated in patients with CAD [4]. There is a sex-specific difference in oxidative stress under different conditions [5, 6]. RAGE appears to be involved in the progression of obesity, associated with inflammation, reactive oxygen species (ROS) production, and insulin sensitivity [7, 8]. Clinical studies confirm sex differences in adipose tissue function, remodeling, and inflammation response [9]. Our recent findings that RAGE-mediated adipose tissue inflammation and insulin signaling are potentially important mechanisms, contributes to the development of obesity-associated insulin resistance [10]. However, sex differences concerning RAGE are not known.

Here, we report that female RAGE knockout mice are protected from high-fat diet (HFD)-induced obesity and adipose tissues-associated insulin signaling. Compared to wild type (WT) mice, RAGE-deficient mice showed reduced body weight gain and improved glucose and insulin tolerance. Furthermore, RAGE deficiency exhibits a reduced M1 polarization in WAT, promotes the adipose-related anti-oxidant genes, and increases the browning of WAT. Our results identify a sex-specific difference for RAGE in regulating adipose tissue-associated insulin resistance.

## Materials and methods

### Animals

RAGE^−/−^ mice were purchased from the Jackson Laboratory (Bar Harbor, ME, USA). All protocols for animal use were reviewed and approved by the Animal Care Committee of Southwest Medical University following Institutional Animal Care and Use Committee guidelines.

### HFD-fed mouse model

Eight-week-old male and female RAGE^−/−^mice were fed a high-fat diet (HFD) (TP2330055A; calories fat 60%, carbohydrate 25%, and protein 15%; Trophic Animal Feed High-tech Co. Ltd, China) for 16 weeks, as described previously [10]. Age-matched male mice that were fed a standard chow diet (ND; TP2330055AC; calories fat 10%, carbohydrate 75%, and protein 15%; Trophic Animal Feed High-tech Co. Ltd, China) were used as controls. Group animal size was *n* = 6–8 per group. The exact group size is specially described in the figure legends.

### Glucose and insulin tolerance tests

Following a 4-h fast, glucose (GTT) and insulin (ITT) tolerance tests were performed in response to intraperitoneal (IP) injection of D-glucose (Roth, Karlsruhe, Germany) (2 g of glucose/kg body mass) or insulin (0.75 U insulin/kg body mass, respectively. Blood samples were obtained from the tail vein, and whole blood glucose levels were measured at 0, 30, 60, and 120 min using a One Touch^®^ Vita^®^ glucometer (Lifescan, Zug, Switzerland).

### Quantitative real-time PCR

Subcutaneous adipose tissue (sAT) was collected and total RNA was extracted using TRIzol reagent (Invitrogen, Carlsbad, CA, USA). RNA samples were pre-treated with deoxyribonuclease I (Invitrogen Life Technologies, Carlsbad, CA, USA), and a SuperScript kit (Invitrogen Life Technologies, Carlsbad, CA, USA) was used to synthesize cDNA according to the manufacturer’s recommendations. qRT-PCR was analyzed using miScript SYBR Green PCR Kits (Qiagen). Levels of macrophage polarization and oxidative stress markers mRNAs were determined using an ABI PRISM 7700 cycler (Applied Biosystems, Foster City, CA). Fold changes in gene expression were determined using the 2 − ΔΔCT method. The values are presented as the mean ± SEM. All primers are listed in Additional file 2: Table S1.

### Immunoblotting

sAT and visceral adipose tissues (epididymal adipose tissues; eAT) lysates were prepared, and equal amounts of protein were subjected to SDS-PAGE and transferred to polyvinylidene difluoride membranes by electroblotting. After blocking, the membranes were incubated with antibodies directed against phospho-Akt (Ser^473^), total AKT (Cell Signaling Technology, Massachusetts, USA). Secondary antibody was horseradish-peroxidase (HRP)-conjugated goat IgG raised against IgG (Santa Cruz Biotechnology). Blots were developed with ECL substrate (Pierce).

### Tissue ROS levels

sAT was isolated, lysed, and the total amount of ROS was determined using the dihydroethidium (DHE) probe according to the manufacturer’s instructions (bjbalb Inc. Beijing, China). All values were normalized to total cellular protein, determined using a BCA assay, and expressed as intensity/mg protein.

### Histological analysis

sAT was isolated, fixed, embedded in paraffin, and serially sectioned (6 µm). Cross-sections were stained with hematoxylin–eosin. The images were captured using a microscope (Nikon). The percentage of positive cells/total adipocytes was quantified in five microscopic fields in each of the three cross sections of each tissue using ImagePro Plus software.

### Statistical analysis

Data are presented as the mean ± SEM of triplicate experiments. The significance of the differences among groups was analyzed by one-way analysis of variance with a post hoc test to determine group differences in the study parameters. All analyses were performed with SPSS software (version 24.0 for Windows; Armonk, NY, USA), and a level of *p* < 0.05 was defined as indicative of statistical significance.

## Results

### Female RAGE deficiency improved glucose and insulin tolerance

**Fig. 1:**
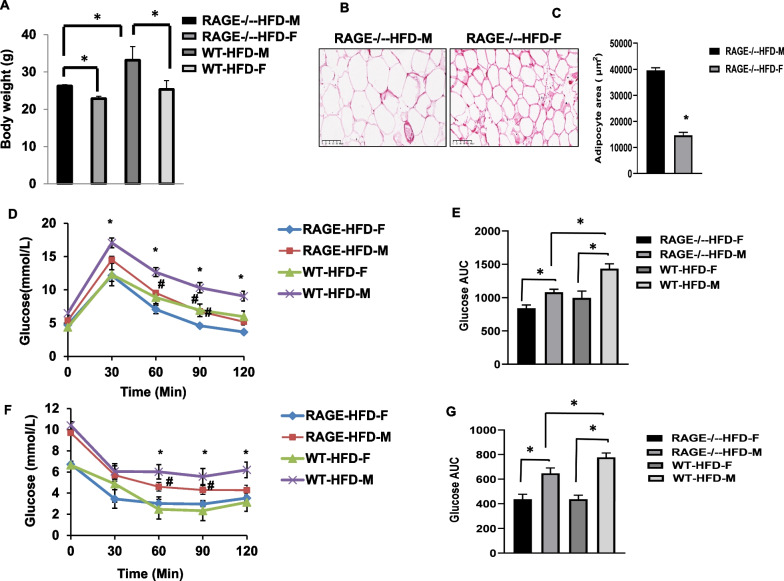
Female RAGE deficiency improved glucose and insulin tolerance. Mice were fed HFD for 16 weeks. **A** Body weight was measured. *n* = 6–8 per group; ^*^p-values indicating significance of difference are indicated in the respective bar diagrams. **B** sAT sections were stained by hematoxylin. Representative histological images were obtained from RAGE^−/−^-HFD mice. Scale bars: 100 μm. sAT, Subcutaneous adipose tissue. **C** The area of adipocyte size is presented as graphs. *n* = 6 per group; *p < *0.05 *vs. male RAGE^−/−^-HFD-M mice. All group data are shown as mean ± SEM. **D, E** Glucose tolerance tests (GTT) and Area under the curve (AUC) in each group. *n* = 6–8 per group. *p < 0.05 vs. female RAGE^−/−^-HFD-F mice. **F, G** Insulin tolerance tests (ITT) and AUC in each group. *n* = 6 per group. *p < 0.05 vs. female WT-HFD-F mice; ^#^*p* < 0.05 vs. female RAGE^−/−^-HFD-F mice. *p-values indicating significance of difference are indicated in the respective bar diagrams

After 16 weeks on the HFD, the body weight of female WT-HFD as well as RAGE-HFD mice was significantly lower as compared to their male counterparts (Fig. 1A). The male WT-HFD mice displayed a significantly increased body weight than male RAGE^−/−^-HFD mice. There is no significant difference in body weight between female WT-HFD and female RAGE^−/−^-HFD mice. sAT adipocyte area was markedly decreased in female RAGE^−/−^-HFD mice compared with male RAGE^−/−^-HFD mice (Fig. 1B, C). Intraperitoneal glucose and insulin tolerance tests were performed to characterize the metabolic state of the animal groups. In WT mice, male WT-HFD feeding caused significantly impaired glucose tolerance and decreased insulin sensitivity compared with female WT-HFD (Fig. 1D–G). Female RAGE^−/−^-HFD mice showed significantly improved glucose and insulin tolerance compared with male RAGE^−/−^-HFD mice, which is similarly happened in normal diet mice (Additional file 1: Fig. S1A,B). Additionally, the male RAGE^−/−^-HFD mice exhibited a significant improvement in GTT and ITT compared with the male WT-HFD mice. However, No differences were found in GTT and ITT between the female WT-HFD and female RAGE^−/−^-HFD mice D mice. These metabolic results suggested that female RAGE deficiency is associated with improved glucose tolerance and insulin sensitivity in HFD-induced obesity in mice.

### Female RAGE deficiency promotes M2 macrophage polarization in adipose tissues

**Fig. 2:**
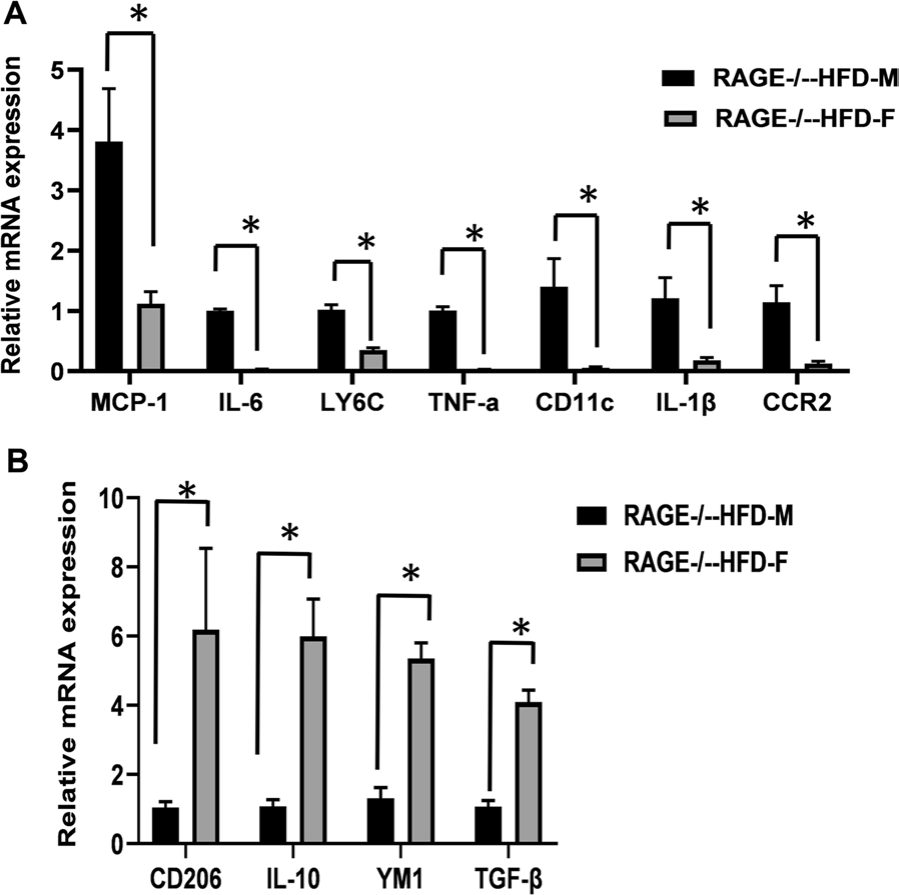
Female RAGE deficiency promotes M2 macrophage polarization in adipose tissues. **A, B** Quantitative RT-PCR analysis of total RNA isolated from sAT for IL-6, IL-1β, TNF-α, MCP-1, and CD11c, IL-10, YM1, TNF-β, and CD206 mRNAs. Data were normalized by the amount of 18 s mRNA and expressed relative to the corresponding male RAGE^−/−^-HFD-M mice. *n* = 6 per group. *p < 0.05 vs. male RAGE^−/−^-HFD-M mice

Since genetic deficiency of RAGE has been previously shown to prevent the effects of HFD on adipose tissue inflammation [8, 10], we next investigated whether the sex differences was involved in the phenotypic switch in macrophages polarization. We evaluated macrophage polarization in sAT by qRT-PCR. As shown in Fig. 2A, the mRNA levels of pro-inflammatory genes, including IL-6, IL-1β, TNF-α, MCP-1, and CD11c, were significantly decreased in sAT from female RAGE^−/−^-HFD mice compared with male RAGE^−/−^-HFD mice, indicating that female RAGE deficiency significantly down-regulates M1 macrophage pro-inflammatory genes. Similarly, compared with male RAGE^−/−^-HFD mice, female RAGE^−/−^-HFD mice exhibited up-regulation of the mRNA levels M2 markers IL-10, YM1, TNF-β, and CD206 (Fig. 2B). These findings support our observation that female RAGE deficiency exhibits an attenuated inflammatory response compared with males due to M2 macrophage polarization.

### Female RAGE deficiency prevents oxidative stress in adipose tissues

**Fig. 3:**
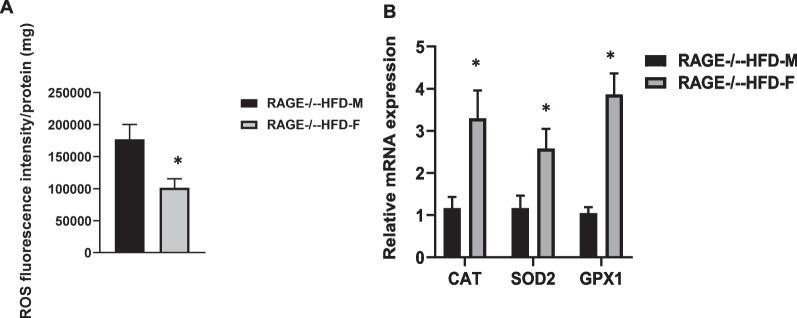
Female RAGE deficiency prevents oxidative stress in adipose tissues. **A** The level of ROS production in sAT from both male and female RAGE^−/−^-HFD mice. **B** CAT, SOD2, and GPX1 mRNA expression levels in sAT of RAGE^−/−^-HFD mice. Data were normalized by the amount of 18 s mRNA and expressed relative to the corresponding male RAGE^−/−^-HFD-M mice. *n* = 6 per group. *p < 0.05. All group data are shown as mean ± SEM

Adipose oxidative stress is a major contributor to metabolic dysfunction and is linked to sex differences [11]. We found that ROS production was decreased in sAT from female RAGE^−/−^-HFD mice compared with RAGE^−/−^-HFD mice (Fig. 3A).The expressions of anti-oxidant genes in sAT were further evaluated by qRT-PCR. The female RAGE^−/−^-HFD significantly increased catalase (CAT)*, *superoxide dismutase 2 (SOD2), and glutathione peroxidase 1 (GPX1) mRNA levels in sAT compared with male RAGE^−/−^-HFD mice. Collectively, these data indicate that female RAGE deficiency could protect obesity-related oxidative stress in adipose tissues compared with male RAGE deficiency.

### Female RAGE deficiency protects insulin-AKT signaling in adipose tissues

**Fig. 4:**
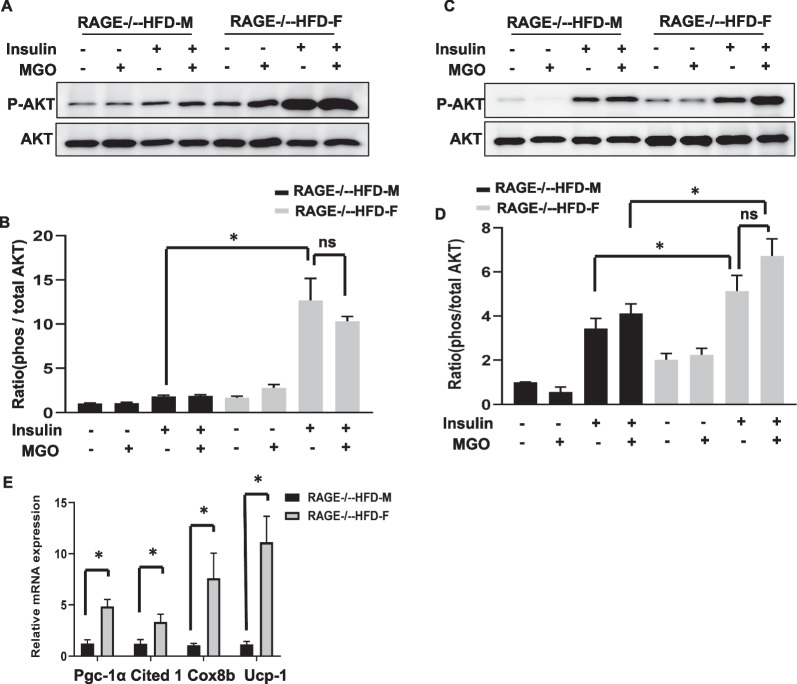
Female RAGE deficiency protects insulin-AKT signaling in adipose tissues. **A, C** The treatment of insulin (100 nM) for 10 min stimulated Ser 473 phosphorylation of AKT after pretreatment with MGO (10 µM) for 16 h in eother sAT (**A**) or eAT (**B**) explants from RAGE^−/−^-HFD mice. Representative immunoblots and quantification from RAGE^−/−^-HFD mice as indicated. **B, D** The graph corresponds to the adjacent blots above and represents densitometric analyses of 3 independent experiments. *p-values indicating significance of difference are indicated in the respective bar diagrams. ns indicates no statistical significance. All group data are shown as mean ± SEM. **C** *Ucp1*, *Pgc1a*, *Cited1*, and *Cox8b* mRNA expression levels in the sAT of male and female RAGE^−/−^-HFD mice. *n* = 6 per group; *p-values indicating significance of difference are indicated in the respective bar diagrams. All group data are shown as mean ± SEM

Methylglyoxal (MGO) is a critical precursor of AGEs, and is associated with the MGO-dependent inhibition of insulin receptor-mediated pathways in adipose tissue [12–14]. Our recent study demonstrated that RAGE mediates insulin sensitivity in WAT explants [10]. We further compared AKT activation responses by the stimulation of insulin (100 nM; 10 min) in sAT explants. The levels of AKT Ser^473^ phosphorylation were significantly higher in both sAT and eAT in female RAGE^−/−^-HFD mice than in male RAGE^−/−^-HFD mice (Fig. 4A–D). However, the fold increase of Ser^473^ AKT phosphorylation were found to be significantly higher in sAT (Fig. 4A) compared with that in eAT (Fig. 4C) in female RAGE^−/−^-HFD mice, and the ratio of female over male in insulin-stimulated Ser^473^ phosphorylation of AKT was markedly less in eAT than in sAT, suggesting there is a sex difference in insulin-stimulated AKT phosphorylation between sAT and eAT. We added the results in revised Fig. 4C, D. MGO could increase AKT signaling in adipose tissues under female RAGE deficiency, not male RAGE deficiency. The pretreatment of MGO (10 µM) for 16 h had no inhibitory effect on insulin-stimulated AKT phosphorylation in both female and male RAGE^−/−^-HDF mice.

The browning of white adipose tissues is associated with increased metabolic rate and improves insulin resistance [15, 16]. We further examined the markers of browning on sAT. As shown in Fig. 4E, female RAGE^−/−^-HFD mice exhibited significantly higher levels of the Ucp1, Pgc1a, Cited1 and Cox8b mRNAs than male RAGE^−/−^-HFD mice on sAT.

## Discussion

Our recent study has reported that AGE–RAGE axis has the potential to impact physiological and pathophysiological metabolic responses in adipose tissues [10]. Interestingly, sex differences were associated with AGE accumulation in T2DM patients [17]. Indeed, adipose tissue function and metabolic syndrome show sex-specific differences [9]. This study aimed to elucidate the sex-specific mechanism of RAGE deficiency in adipose tissue metabolic regulation and systemic glucose homeostasis using male and female RAGE^−/−^ mice fed HFD diet.

There are major sex differences in insulin sensitivity and glucose metabolism in adipose tissue regulated by physiological levels of sex steroids [18]. Estrogen stimulates the expression of RAGE and is controlled mainly via estrogen receptor alpha and RAGE-dependent signaling [19, 20]. Soluble form of RAGE (sRAGE), which acts as a decoy for AGE, has been correlated with T2DM patients. It has been noted that plasma sRAGE levels were directly correlated with sex-dependent BMI, waist/hip circumference ratio, and fasting glycemia between obese and non-obese individuals [21]. Accumulation of adipose tissue macrophage is strongly associated with insulin resistance. Our recent study has suggested that RAGE is involved in the development of macrophage recruitment and polarization in adipose tissues in obese [10]. In this study, after 16 weeks of HF feeding, qRT-PCR analysis showed decreased adipose tissue gene expression of M1 polarization markers in female RAGE^−/−^ mice. Further studies are required to clarify how macrophage-mediated chronic inflammation in WAT contributes to insulin resistance in female RAGE^−/−^ mice.

Our previous report demonstrated that RAGE deficiency improved insulin sensitivity and glucose tolerance [10]. Under HF dietary conditions, female RAGE^−/−^ mice showed significantly improved glucose tolerance than male RAGE^−/−^ mice. Furthermore, body weight and adipocyte size in female RAGE^−/−^ mice were less significant than in male RAGE^−/−^ mice. These different responses to HFD feeding between male and female RAGE^−/−^ mice are likely related to the secretion of estrogen and sex-specific effects of sex hormones on adipose tissue distribution [22, 23].

The AKT signaling plays an essential role in glucose homeostasis mediated by insulin*,* and *o*besity-mediated glucose metabolism reduces insulin-AKT phosphorylation. Our recent finding that insulin-induced AKT phosphorylation was impaired in adipose tissue from male RAGE^−/−^-HFD mice compared with WT-HFD mice [10]. The action of RAGE expression likely appears tissue-specific insulin sensitivity on a high-fat diet. In the current study, female RAGE^−/−^-HFD mice exhibit a significantly higher level of AKT phosphorylation in sAT than male RAGE^−/−^-HFD, which could account for the improvement of glucose homeostasis.

Browning of white adipose tissue appears to positively affect energy expenditure, adiposity, and glucose homeostasis [16, 24]. Our previous report addressed that RAGE deficiency exhibits the browning of white adipose tissue under HF dietary conditions. In this study, in female RAGE^−/−^ mice, we observed markedly increased mRNA expressions of genes for thermogenesis regulators, Ucp-1 and Pgc1a, mitochondrial component, cox8b, and beige adipocyte marker, cited1. Ucp-1 has been demonstrated to protect from oxidative stress by inhibiting mitochondrial ROS production [25, 26]. Our study found that male RAGE^−/−^ mice had a higher ROS production in adipose tissues than female RAGE^−/−^ mice, suggesting that RAGE-regulated browning of adipose tissue is a female-specific mechanism underlying insulin resistance improvement. The relationship between RAGE, sex hormones, and oxidative stress is needed to elucidate the sex-specific effects of RAGE depletion.

### Perspectives and significance

In summary, we found that the protective effect of RAGE deficiency from obesity-induced glucose homeostasis is sex-specific. Female RAGE-deficient mice had markedly improved glucose and insulin tolerance, insulin-AKT signaling, which was associated with a down-regulated M1 macrophage pro-inflammatory genes, an increase in anti-oxidant genes, and browning of subcutaneous adipose tissue. Our findings suggest that sex-specific dimorphic pattern plays an important role for RAGE in obesity-induced adipose tissue insulin resistance.

## Supplementary Information

**Additional file 1: Figure S1.** Female RAGE deficiency improved glucose and insulin tolerance in normal diet mice. (A) Glucose tolerance tests (GTT) and Area under the curve (AUC) in each group. *n* = 6 per group. *p < 0.05 vs. female RAGE^−/−^-HFD-F mice; ^#^*p* < 0.05 vs. female RAGE^−/−^-ND-F mice. (B) Insulin tolerance tests (ITT) and AUC in each group. *n* = 6 per group. *p < 0.05 vs. female RAGE^−/−^-HFD-F mice; ^#^*p* < 0.05 vs. female RAGE^−/−^-ND-F mice. ND; normal diet. All group data are shown as mean ± SEM.

**Additional file 2: Table S1.** Sequences of primers used in the study.

## Data Availability

The data sets used during the current study are available from the corresponding author upon reasonable request.

## Abbreviations

CAT: Catalase
GPX1: Glutathione peroxidase 1
HFD: High-fat diet
MGO: Methylglyoxal
RAGE: Receptor for advanced glycation end products
ROS: Reactive oxygen species
SOD2: Superoxide dismutase 2
sAT: Subcutaneous adipose tissue
WAT: White adipose tissue
