# Supplementary material for: RAGE displays sex-specific differences in obesity-induced adipose tissue insulin resistance
Source: Biol Sex Differ. 2022 Nov 8;13:65. doi: 10.1186/s13293-022-00476-6 (PMC9641909; doi:10.1186/s13293-022-00476-6)
Supplement: Supplementary file 1 — Additional file 1: Figure S1. Female RAGE deficiency improved glucose and insulin tolerance in normal diet mice. (A) Glucose tolerance tests (GTT) and Area under the curve (AUC) in each group. n = 6 per group. *p < 0.05 vs. female RAGE−/−-HFD-F mice; #p < 0.05 vs. female RAGE−/−-ND-F mice. (B) Insulin tolerance tests (ITT) and AUC in each group. n = 6 per group. *p < 0.05 vs. female RAGE−/−-HFD-F mice; #p < 0.05 vs. female RAGE−/−-ND-F mice. ND; normal diet. All group data are shown as mean ± SEM. [file 13293_2022_476_MOESM1_ESM.pptx]

## Slide 1
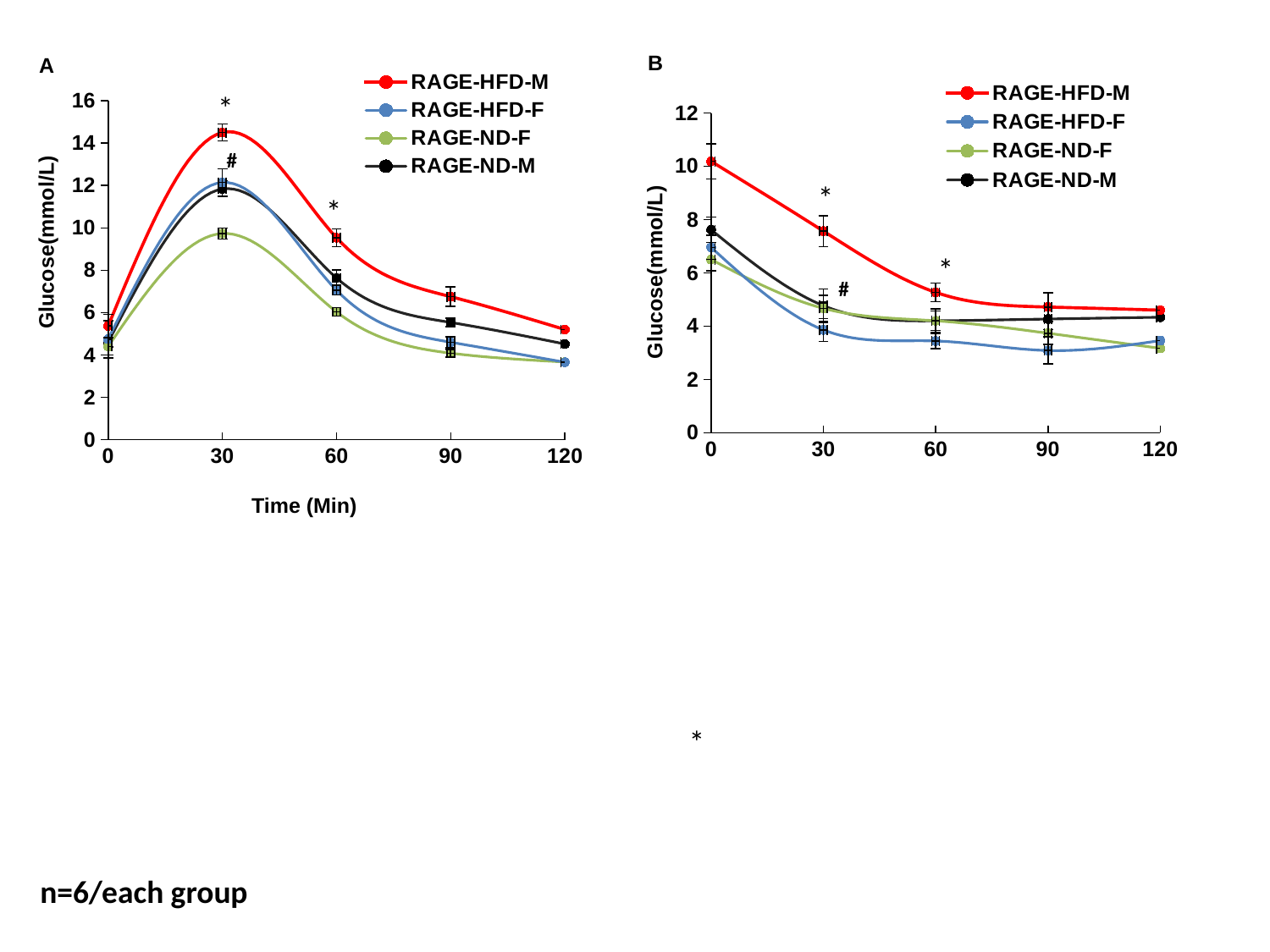

B
A
### Chart
| Category | RAGE-HFD-M | RAGE-HFD-F | | |
|---|---|---|---|---|
### Chart
| Category | RAGE-HFD-M | RAGE-HFD-F | | |
|---|---|---|---|---|*
#
*
*
Glucose(mmol/L)
*
Glucose(mmol/L)
#
Time (Min)
*
n=6/each group
