# Supplementary material for: RAGE displays sex-specific differences in obesity-induced adipose tissue insulin resistance
Source: Biol Sex Differ. 2022 Nov 8;13:65. doi: 10.1186/s13293-022-00476-6 (PMC9641909; doi:10.1186/s13293-022-00476-6)
Supplement: Supplementary file 2 — Additional file 2: Table S1. Sequences of primers used in the study. [file 13293_2022_476_MOESM2_ESM.docx]

# Table 1 Sequences of primers used in the study

| Gene  (Accession number) |  | Primer |
| --- | --- | --- |
| CCR2  (NM_009915) | Forward  Reverse | GCAAGTTCAGCTGCCTGCAA  ATGCCGTGGATGAACTGAGGTAA |
| Ly6C  (NM_001252058) | Forward  Reverse | ACTGTGCCTGCAACCTTGTC  CACACAGTAGGGCCACAAGA |
| IL-6  (NM_031168) | Forward  Reverse | CACATGTTCTCTGGGAAATCG  TTGTATCTCTGGAAGTTTCAGATTGTT |
| TNF-α  (NM_001124357) | Forward  Reverse | ACGGCATGGATCTCAAAGAC  AGATAGCAAATCGGCTGACG |
| CD11c | Forward | CACTCAGTGACTGCCCAAAA |
| ([NM_021334.2](https://www.ncbi.nlm.nih.gov/entrez/viewer.fcgi?db=nucleotide&id=118130485)) | Reverse | CCTCAAGACAGGACATCGCT |
| IL-1β | Forward | ACTACAGGCTCCGAGATGAACAAC |
| ([NM_008361.4](https://www.ncbi.nlm.nih.gov/entrez/viewer.fcgi?db=nucleotide&id=921274059)) | Reverse | CCCAAGGCCACAGGTATTTT |
| MCP1  (NM_011333.3) | Forward  Reverse | ACGCAGGTCCCTGTCATG  GTTCACTGTCACACTGGTCA |
| Ym1   NM_009892.3 | Forward  Reverse | AGAGTGCTGATCTCAATGTGG  GGGCACCAATTCCAGTCTTAG |
| CAT   NM_080483.3 | Forward  Reverse | TTACCCCAACAGCTTCAGCG  GTCCGCACCTGAGTGACATT |
| SOD2   NM_013671.3 | Forward  Reverse | GAGAACCCAAAGGAGAGTTGC  CTTATTGAAGCCAAGCCAGCC |
| GPX1   NM_001329528.1 | Forward  Reverse | TTCGGACACCAGGAGAATGG  TAAAGAGCGGGTGAGCCTTC |
| IL-10 | Forward | TGTCAAATTCATTCATGGCCT |
| ([NM_010548.2](https://www.ncbi.nlm.nih.gov/entrez/viewer.fcgi?db=nucleotide&id=291575143)) | Reverse | ATCGATTTCTCCCCTGTGAA |
| TGFβ1 | Forward | TGCTAATGGTGGACCGCAA |
| ([NM_011577.2](https://www.ncbi.nlm.nih.gov/entrez/viewer.fcgi?db=nucleotide&id=930697458)) | Reverse | CACTGCTTCCCGAATGTCTGA |
| CD206 | Forward | CATGGATGTTGATGGCTACTGGAG |
| ([NM_008625.2](https://www.ncbi.nlm.nih.gov/entrez/viewer.fcgi?db=nucleotide&id=224967061)) | Reverse | GTCTGTTCTGACTCTGGACACTTG |
| 18S rRNA | Forward | GCAATTATTCCCCATGAACG |
| (NR_003278.3) | Reverse | GGCCTCACTAAACCATCCAA |
